# Supplementary material for: Resolving the full spectrum of human genome variation using Linked-Reads
Source: Genome Res. 2019 Apr;29(4):635–45. doi: 10.1101/gr.234443.118 (PMC6442396; doi:10.1101/gr.234443.118)
Supplement: Supplemental Material [file supp_gr.234443.118_Supplemental_longranger-2.2.zip › longranger-2.2.tar.bz2!/lr2.2-public/lib/go/src/code.google.com/vitess/docs/user-guide/horizontal-sharding.html]

Vitess / Horizontal Sharding (Tutorial, manual)


Toggle navigation

- Overview
- Guides
- Reference
- Blog
- GitHub

- #### Overview

  - What is Vitess
  - Scaling MySQL with Vitess
  - Key Concepts
- #### Getting Started

  - Run Vitess on Kubernetes
    - Custom Docker Build
  - Run Vitess Locally
- #### User Guide

  - Introduction- Client Libraries- Backing Up Data- Reparenting
        - Schema Management
        - VSchema Guide
        - Schema Swap (Tutorial)
        - Sharding
          - Horizontal Sharding (Tutorial, automated)
          - Horizontal Sharding (Tutorial, manual)
          - Sharding in Kubernetes (Tutorial, automated)
          - Sharding in Kubernetes (Tutorial, manual)
        - Vitess Sequences
        - MySQL Server Protocol
        - Vitess and Replication
        - Update Stream
        - Row Based Replication
        - Topology Service
        - Transport Security Model
        - Launching
          - Scalability Philosophy
          - Production Planning
          - Server Configuration
          - 2PC Guide
          - Troubleshooting
        - Upgrading
- #### Reference Guides

  - Vitess API- vtctl Commands
- #### Other Resources

  - Presentations- Blog- Roadmap
- #### Contributing

  - Contributing to Vitess- GitHub Workflow- Code Reviews
- GitHub

# Horizontal Sharding (Tutorial, manual)

- #### Overview

  - What is Vitess
  - Scaling MySQL with Vitess
  - Key Concepts
- #### Getting Started

  - Run Vitess on Kubernetes
    - Custom Docker Build
  - Run Vitess Locally
- #### User Guide

  - Introduction- Client Libraries- Backing Up Data- Reparenting
        - Schema Management
        - VSchema Guide
        - Schema Swap (Tutorial)
        - Sharding
          - Horizontal Sharding (Tutorial, automated)
          - Horizontal Sharding (Tutorial, manual)
          - Sharding in Kubernetes (Tutorial, automated)
          - Sharding in Kubernetes (Tutorial, manual)
        - Vitess Sequences
        - MySQL Server Protocol
        - Vitess and Replication
        - Update Stream
        - Row Based Replication
        - Topology Service
        - Transport Security Model
        - Launching
          - Scalability Philosophy
          - Production Planning
          - Server Configuration
          - 2PC Guide
          - Troubleshooting
        - Upgrading
- #### Reference Guides

  - Vitess API- vtctl Commands
- #### Other Resources

  - Presentations- Blog- Roadmap
- #### Contributing

  - Contributing to Vitess- GitHub Workflow- Code Reviews

This guide walks you through the process of sharding an existing unsharded
Vitess keyspace.

## Prerequisites

We begin by assuming you've completed the
Getting Started guide,
and have left the cluster running.

## Overview

The sample clients in the `examples/local` folder use the following schema:

```
CREATE TABLE messages (
  page BIGINT(20) UNSIGNED,
  time_created_ns BIGINT(20) UNSIGNED,
  message VARCHAR(10000),
  PRIMARY KEY (page, time_created_ns)
) ENGINE=InnoDB
```

The idea is that each page number represents a separate guestbook in a
multi-tenant app. Each guestbook page consists of a list of messages.

In this guide, we'll introduce sharding by page number.
That means pages will be randomly distributed across shards,
but all records for a given page are always guaranteed to be on the same shard.
In this way, we can transparently scale the database to support arbitrary growth
in the number of pages.

## Configure sharding information

The first step is to tell Vitess how we want to partition the data.
We do this by providing a VSchema definition as follows:

```
{
  "sharded": true,
  "vindexes": {
    "hash": {
      "type": "hash"
    }
  },
  "tables": {
    "messages": {
      "column_vindexes": [
        {
          "column": "page",
          "name": "hash"
        }
      ]
    }
  }
}
```

This says that we want to shard the data by a hash of the `page` column.
In other words, keep each page's messages together, but spread pages around
the shards randomly.

We can load this VSchema into Vitess like this:

```
vitess/examples/local$ ./lvtctl.sh ApplyVSchema -vschema "$(cat vschema.json)" test_keyspace
```

## Bring up tablets for new shards

In the unsharded example, you started tablets for a shard
named *0* in *test\_keyspace*, written as *test\_keyspace/0*.
Now you'll start tablets for two additional shards,
named *test\_keyspace/-80* and *test\_keyspace/80-*:

```
vitess/examples/local$ ./sharded-vttablet-up.sh
```

Since the sharding key is the page number,
this will result in half the pages going to each shard,
since *0x80* is the midpoint of the
sharding key range.

These new shards will run in parallel with the original shard during the
transition, but actual traffic will be served only by the original shard
until we tell it to switch over.

Check the *vtctld* web UI, or the output of `lvtctl.sh ListAllTablets test`,
to see when the tablets are ready. There should be 5 tablets in each shard.

Once the tablets are ready, initialize replication by electing the first master
for each of the new shards:

```
vitess/examples/local$ ./lvtctl.sh InitShardMaster -force test_keyspace/-80 test-0000000200
vitess/examples/local$ ./lvtctl.sh InitShardMaster -force test_keyspace/80- test-0000000300
```

Now there should be a total of 15 tablets, with one master for each shard:

```
vitess/examples/local$ ./lvtctl.sh ListAllTablets test
### example output:
# test-0000000100 test_keyspace 0 master 10.64.3.4:15002 10.64.3.4:3306 []
# ...
# test-0000000200 test_keyspace -80 master 10.64.0.7:15002 10.64.0.7:3306 []
# ...
# test-0000000300 test_keyspace 80- master 10.64.0.9:15002 10.64.0.9:3306 []
# ...
```

## Copy data from original shard

The new tablets start out empty, so we need to copy everything from the
original shard to the two new ones, starting with the schema:

```
vitess/examples/local$ ./lvtctl.sh CopySchemaShard test_keyspace/0 test_keyspace/-80
vitess/examples/local$ ./lvtctl.sh CopySchemaShard test_keyspace/0 test_keyspace/80-
```

Next we copy the data. Since the amount of data to copy can be very large,
we use a special batch process called *vtworker* to stream the data from a
single source to multiple destinations, routing each row based on its
*keyspace\_id*:

```
vitess/examples/local$ ./sharded-vtworker.sh SplitClone test_keyspace/0
### example output:
# I0416 02:08:59.952805       9 instance.go:115] Starting worker...
# ...
# State: done
# Success:
# messages: copy done, copied 11 rows
```

Notice that we've only specified the source shard, *test\_keyspace/0*.
The *SplitClone* process will automatically figure out which shards to use
as the destinations based on the key range that needs to be covered.
In this case, shard *0* covers the entire range, so it identifies
*-80* and *80-* as the destination shards, since they combine to cover the
same range.

Next, it will pause replication on one *rdonly* (offline processing) tablet
to serve as a consistent snapshot of the data. The app can continue without
downtime, since live traffic is served by *replica* and *master* tablets,
which are unaffected. Other batch jobs will also be unaffected, since they
will be served only by the remaining, un-paused *rdonly* tablets.

## Check filtered replication

Once the copy from the paused snapshot finishes, *vtworker* turns on
filtered replication
from the source shard to each destination shard. This allows the destination
shards to catch up on updates that have continued to flow in from the app since
the time of the snapshot.

When the destination shards are caught up, they will continue to replicate
new updates. You can see this by looking at the contents of each shard as
you add new messages to various pages in the Guestbook app. Shard *0* will
see all the messages, while the new shards will only see messages for pages
that live on that shard.

```
# See what's on shard test_keyspace/0:
vitess/examples/local$ ./lvtctl.sh ExecuteFetchAsDba test-0000000100 "SELECT * FROM messages"
# See what's on shard test_keyspace/-80:
vitess/examples/local$ ./lvtctl.sh ExecuteFetchAsDba test-0000000200 "SELECT * FROM messages"
# See what's on shard test_keyspace/80-:
vitess/examples/local$ ./lvtctl.sh ExecuteFetchAsDba test-0000000300 "SELECT * FROM messages"
```

You can run the client script again to add some messages on various pages
and see how they get routed.

## Check copied data integrity

The *vtworker* batch process has another mode that will compare the source
and destination to ensure all the data is present and correct.
The following commands will run a diff for each destination shard:

```
vitess/examples/local$ ./sharded-vtworker.sh SplitDiff test_keyspace/-80
vitess/examples/local$ ./sharded-vtworker.sh SplitDiff test_keyspace/80-
```

If any discrepancies are found, they will be printed.
If everything is good, you should see something like this:

```
I0416 02:10:56.927313      10 split_diff.go:496] Table messages checks out (4 rows processed, 1072961 qps)
```

## Switch over to new shards

Now we're ready to switch over to serving from the new shards.
The MigrateServedTypes
command lets you do this one
tablet type at a time,
and even one cell
at a time. The process can be rolled back at any point *until* the master is
switched over.

```
vitess/examples/local$ ./lvtctl.sh MigrateServedTypes test_keyspace/0 rdonly
vitess/examples/local$ ./lvtctl.sh MigrateServedTypes test_keyspace/0 replica
vitess/examples/local$ ./lvtctl.sh MigrateServedTypes test_keyspace/0 master
```

During the *master* migration, the original shard master will first stop
accepting updates. Then the process will wait for the new shard masters to
fully catch up on filtered replication before allowing them to begin serving.
Since filtered replication has been following along with live updates, there
should only be a few seconds of master unavailability.

When the master traffic is migrated, the filtered replication will be stopped.
Data updates will be visible on the new shards, but not on the original shard.
See it for yourself: Add a message to the guestbook page and then inspect
the database content:

```
# See what's on shard test_keyspace/0
# (no updates visible since we migrated away from it):
vitess/examples/local$ ./lvtctl.sh ExecuteFetchAsDba test-0000000100 "SELECT * FROM messages"
# See what's on shard test_keyspace/-80:
vitess/examples/local$ ./lvtctl.sh ExecuteFetchAsDba test-0000000200 "SELECT * FROM messages"
# See what's on shard test_keyspace/80-:
vitess/examples/local$ ./lvtctl.sh ExecuteFetchAsDba test-0000000300 "SELECT * FROM messages"
```

## Remove original shard

Now that all traffic is being served from the new shards, we can remove the
original one. To do that, we use the `vttablet-down.sh` script from the
unsharded example:

```
vitess/examples/local$ ./vttablet-down.sh
```

Then we can delete the now-empty shard:

```
vitess/examples/local$ ./lvtctl.sh DeleteShard -recursive test_keyspace/0
```

You should then see in the vtctld **Topology** page, or in the output of
`lvtctl.sh ListAllTablets test` that the tablets for shard *0* are gone.

## Tear down and clean up

Since you already cleaned up the tablets from the original unsharded example by
running `./vttablet-down.sh`, that step has been replaced with
`./sharded-vttablet-down.sh` to clean up the new sharded tablets.

```
vitess/examples/local$ ./vtgate-down.sh
vitess/examples/local$ ./sharded-vttablet-down.sh
vitess/examples/local$ ./vtctld-down.sh
vitess/examples/local$ ./zk-down.sh
```

Contact: vitess@googlegroups.com  **·**  
Announcements  **·**  
© 2017 Vitess powered by Google Inc
